# Sex differences in guideline adherence for coronary angiography in patients with suspected chronic coronary syndrome in Germany: insights from the ENLIGHT-KHK trial

**DOI:** 10.1007/s00392-025-02655-y

**Published:** 2025-05-06

**Authors:** Hannah Kentenich, Arim Shukri, Dirk Müller, Bastian Wein, Oliver Bruder, Stephanie Stock, Yana Kampfer

**Affiliations:** 1 Faculty of Medicine and University Hospital Cologne, Institute for Health Economics and Clinical Epidemiology, University of Cologne, Gleueler Straße 176-178 / II, 50935 Cologne, Germany; 2 Contilia Heart and Vascular Centre, Elisabeth-Hospital, Klara-Kopp-Weg 1, 45138 Essen, Germany; 3 Faculty of Medicine, Cardiology, University of Augsburg, Stenglinstrasse 2, 86156 Augsburg, Germany; 4 Faculty of Medicine, Ruhr University Bochum, 44801 Bochum, Germany

**Keywords:** Coronary artery disease, Coronary angiography, Sex differences, Guideline adherence

## Abstract

**Background:**

For the management of acute coronary syndrome, literature shows lower healthcare providers’ guideline adherence for women than for men. Since less is known about the management of chronic coronary syndrome (CCS), this study investigated patient-related sex differences in providers’ guideline adherence for invasive coronary angiography (CA) performed in patients with suspected CCS.

**Methods:**

Using data from the German ENLIGHT-KHK trial, patients with suspected CCS who underwent a CA were analysed. To assess the association between patient sex and physicians’ adherence to the German National Disease Management Guideline “Chronic coronary artery disease” of 2019, binary logistic regression models were developed. Covariates included age, symptoms, risk factors, comorbidities, and non-invasive testing and its results. To examine sex differences in predictors of guideline adherence, models were run separately for women and men.

**Results:**

Two hundred seventy-three women and three hundred eighty-six men were included (aged 67 ± 10 years). Physicians’ guideline adherence for CA was lower for women than for men (19.4% vs. 30.1%, *p* = 0.002). CAs were less likely to be guideline-adherent for women with suspected CCS than men (OR 0.4, *p* < 0.05). Guideline adherence predictors differed between women and men. For example, men’s predictors included non-invasive testing and its results, age, typical angina and smoking; of these, only a positive non-invasive test result had an impact for women.

**Conclusion:**

Our results indicate a less guideline-adherent diagnostic workup of CA for women with suspected CCS than men. This might reflect a limited awareness of CCS in women and insufficiently sex-specific guideline recommendations.

**Trial registration:**

German Clinical Trials Register DRKS00015638, Registered February 19, 2019; Universal Trial Number (UTN): U1111-1227-8055.

**Graphical abstract:**

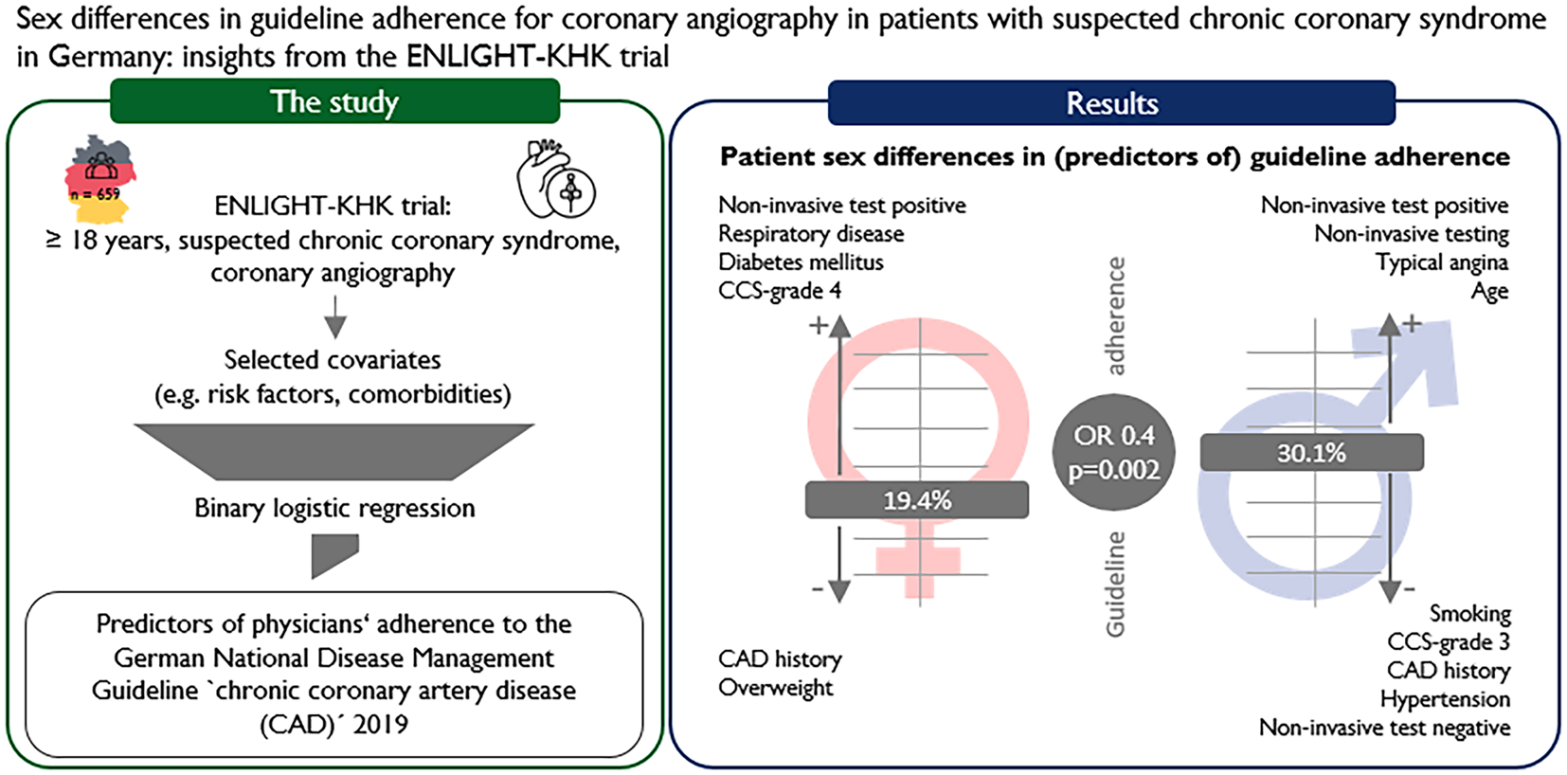

**Supplementary Information:**

The online version contains supplementary material available at 10.1007/s00392-025-02655-y.

## Background

Coronary artery disease (CAD) is the leading cause of death in Germany [1] for both women and men (2020: 86 and 179 deaths per 100,000 inhabitants) [2]. To reduce this high mortality rate, appropriate management is important. National and international clinical practice guidelines therefore provide evidence-based recommendations on the diagnosis and treatment of CAD, for both the acute (acute coronary syndrome (ACS)) and the chronic (chronic coronary syndrome (CCS)) presentations of the disease [3–6].

In particular, an evidence-based diagnosis of CCS and the resulting prompt therapy might contribute to a reduction of morbidity and mortality and an improvement in quality of life among CAD patients [4, 6]. For diagnosing CCS, invasive coronary angiography (CA) is regarded as the reference standard [7]. However, CA as an invasive procedure is only recommended for patients considered for revascularization [4, 6]. Thus, an appropriate selection of eligible patients is essential. In the German National Disease Management Guideline (GNDMG) “Chronic CAD” of 2019, an algorithmic approach recommends a CA either for patients with an intermediate pre-test probability (PTP) of obstructive CAD (15–85%) and positive results in anatomical (coronary computed tomography angiography (CCTA)) or functional non-invasive testing (e.g. stress echocardiography (ECHO) or cardiac magnetic resonance imaging (CMR)), or for patients with a high PTP (> 85%) [4]. The PTP is estimated based on age, sex and the patient’s symptoms [4, 8, 9]. The 2019 European Society of Cardiology (ESC) guidelines on the diagnosis and management of CCS recommend a non-invasive test as an initial step for all patients for whom CAD cannot be excluded by clinical assessment [6].

There is increasing evidence from OECD countries that, in the management of ACS and the medical therapy for CCS, healthcare providers’ adherence to the guidelines is lower for women than for men [10–14]. This might contribute to the higher risk of death and acute myocardial infarction among women with CAD compared to men [10, 12]. In contrast, little is known about patient-related sex differences in the guideline-adherent use of CA for suspected CCS [15].

For women with suspected CCS, a lower guideline adherence for CA increases the risk of being underdiagnosed (in cases of no CA despite indication) or exposed more often to the burden and risk of CA [16] (in cases of CA without indication) compared to men [4]. This could result in a higher morbidity and mortality and a reduced quality of life for women than compared to men.

This study aims to gain initial insights into sex differences in the guideline adherence for CA for suspected CCS in Germany. Using data from patients with suspected CCS who received a CA, two research questions were investigated: i) Is there a difference between the healthcare providers’ guideline adherence for the use of CA in women with suspected CCS compared to men?, ii) Do the predictors affecting healthcare providers’ guideline adherence differ between women and men with suspected CCS?

## Methods

### Study cohort

This study is a prespecified subanalysis of the German ENLIGHT-KHK trial, a multicentre, prospective, observational study that investigated the nature and extent of guideline adherence in the diagnostic workup of CA in patients with suspected obstructive CCS and evaluated the clinical and economic consequences of potential guideline non-adherence [17].

The ENLIGHT-KHK trial (2019–2021) included 901 adults who presented with suspected obstructive CCS with excluded ACS (i.e. unstable angina or acute myocardial infarction) to one of nine non-university hospitals in Germany, and who were assigned to or underwent a CA within the current admission [17]. Patients were included in different clinical settings and during different steps of the diagnostic workup, thus resulting in 5 cohorts [17]. These were: (1) patients referred for elective CA [18], (2) patients presenting at the emergency department who underwent CA [19], (3) patients presenting in the outpatient department without prior diagnostic workup, (4) patients presenting at the emergency department undergoing planned non-invasive testing, and (5) patients referred for elective, non-invasive image testing. This analysis included all patients of the ENLIGHT-KHK trial in whom adherence to the GNDMG “Chronic CAD” of 2019 was assessed, i.e. those with CA and sufficient data on PTP, symptoms and non-invasive test results. Of 695 patients with CA, guideline adherence could not be assessed in 36 patients due to missing data on angina type and non-invasive test results, leading to a total of 659 patients who were included in this analysis [17, 20].

### Study outcomes and data collection

To answer research question i), the difference in physicians’ adherence to the GNDMG “Chronic CAD” of 2019 for CA between women and men was evaluated, together with the association between patient sex (i.e. self-reported biological sex [21]) and physicians’ guideline adherence. In line with research question ii), the extent to which factors influencing physicians’ guideline adherence for CA differed between women and men was investigated. For this purpose, the number, type, effect and impact of the predictors of guideline adherence were compared between women and men.

All the data were taken from the ENLIGHT-KHK trial (Table 1) [17, 20]. Guideline adherence was defined according to the GNDMG [4] and was evaluated using a priori defined rules based on data from patients’ medical records and patients’ questionnaires. These data included the patient’s PTP [8, 9] for having an obstructive CAD and the results of the prior non-invasive testing (i.e. exercise electrocardiogram (ECG), stress ECHO, myocardial perfusion scintigraphy (MPS), CCTA and stress-CMR). PTP was calculated using the age and sex obtained from patients’ medical records, while the type of anginal symptoms was obtained from a standardized patient questionnaire. Non-invasive testing and its results were obtained from patients’ medical records [17]. For the rationale used to define guideline adherence, see Table 2 [20].

**Table 1:** Data sources and variables

Data source	Variables
Patients’ medical record	Demographics: Sex Age Risk factors: Hypertension Diabetes mellitus^a^ Smoking^b^ Overweight^c^ Hypercholesterolaemia Family history of CAD Comorbidities: CAD history^d^ Chronic renal failure^e^ Respiratory disease^f^ Peripheral artery disease Depression Non-invasive testing: Exercise electrocardiogram Stress echocardiography Coronary computed tomography angiography Stress cardiac magnetic resonance imaging Myocardial perfusion scintigraphy Non-invasive test result^g^
Patient questionnaires	Type of anginal symptoms^h^ Symptom severity^i^
Analysis results	Guideline adherence

*CAD* coronary artery disease

^a^Type 1 or 2

^b^Ever/never

^c^BMI ≥ 25

^d^Known CAD, myocardial infarction, catheterization without stent, revascularization

^e^Defined as an estimated glomerular filtration rate < 60 ml/min/1.72 m^2^

^f^Chronic obstructive lung disease or other

^g^Positive (at least one test positive), negative (no test positive and at least one test negative), unclear (no test positive/negative and at least one test unclear)

^h^Typical angina, atypical angina, non-anginal chest pain

^i^Canadian Cardiovascular Society (CCS)-Grade 0–4

**Table 2:** Definition of guideline adherence

Pre-test probability	Non-invasive testing^a^	Guideline adherence of coronary angiography
Low (< 15%)	Not done Non-pathological result Pathological result Inconclusive result	No No Yes Yes
Intermediate (15–85%)	Not done Non-pathological result Pathological result Inconclusive result	No No Yes Yes
High (≥ 85%)	Irrespective of non-invasive testing	Yes

^a^Stress echocardiography, coronary computed tomography angiography, myocardial perfusion scintigraphy or cardiac stress magnetic resonance imaging

Furthermore, patient demographics, risk factors and comorbidities were obtained from patients’ medical records, and data on the severity of symptoms were collected using a standardized patient questionnaire [17].

### Statistical analyses

Baseline characteristics of the study population were reported using mean and standard deviation (SD) for continuous variables, and numbers and proportions for categorical variables. Characteristics were compared between women and men using a Chi-square test for categorical variables and a Mann–Whitney *U* test for continuous and ordinal variables. To assess the correlation between the PTP and obstructive CAD, Spearman’s rank correlation coefficient (ρ) was calculated.

To answer research question i), firstly, a Chi-square test was conducted comparing the physicians’ guideline adherence between both patient sexes. The results were presented as difference with a corresponding 95% confidence interval and *p* value. Secondly, a multiple binary logistic regression model was developed to investigate the adjusted association between patient sex and physicians’ guideline adherence. Factors that might influence physicians’ decision-making on CA use were determined based on the literature and guidelines [4, 22, 23] and were verified by a clinical expert (BW). No interaction terms were included, since they would increase the complexity and reduce the interpretability of the models. The final set of covariates included age, type of anginal symptoms, symptom severity according to Canadian Cardiovascular Society classification (CCS-Grade), risk factors, comorbidities, non-invasive testing (i.e. at least one non-invasive test) and the test result (Model 1) as defined in Table 1. Since the ENLIGHT-KHK trial determined guideline adherence based on the results of prior non-invasive testing (Table 2) [17, 20], variables including non-invasive testing and its result were expected to have a large influence on our analysis. To account for this and to analyse the change in explanatory power, a second regression model was fitted; this adjusted for the aforementioned covariates with the exception of non-invasive testing and its result (Model 2).

To answer research question ii), patient sex-specific predictors of physicians’ guideline adherence were examined and compared by running both regression models separately for women (Model 1-f, Model 2-f) and men (Model 1-m, Model 2-m).

The models were developed by backward stepwise regression (details on the analysis are presented in Online Resource, Text S1). Regression model results were presented as an odds ratio (OR) with a corresponding 95% confidence interval and *p* value. Model performance was assessed using the pseudo-R^2^ (Nagelkerke R^2^) [24]. For all analyses, a *p* value < 0.05 indicated statistical significance. To account for multiple testing, the Benjamini–Hochberg procedure was used. All analyses were conducted with IBM SPSS Statistics for Windows, version 29, Armonk, NY: IBM Corp.

## Results

### Patient characteristics

A total of 659 patients were included, whose baseline characteristics are summarized in Table 3. On average, the patients were 67 ± 10 years old. The majority of the patients (n = 495, 75%) had three or more risk factors, with hypertension (n = 549, 83%) and overweight (n = 519, 79%) being the most common. More than one third of the patients presented with typical angina (n = 224, 34%) or received non-invasive testing prior to CA (n = 237, 36%). 273 patients (41%) were female and 386 (59%) were male. The women were older and less likely to be a current or former smoker or have a history of CAD (*p* < 0.001). Furthermore, the women tended to be less likely to receive non-invasive testing (*p* < 0.05 without Benjamini–Hochberg adjustment, see Online Resource, Table S1). CA identified obstructive CAD in 51% of women and 74% of men. The correlation between PTP and obstructive CAD was significant for men (*ρ* = 0.135, *p* = 0.008) but not for women (*ρ* = 0.082, *p* > 0.05).

**Table 3:** Baseline characteristics and coronary angiography results of participants

Characteristic	Total (n = 659)	Women (n = 273)	Men (n = 386)	*p* value^a^
Age (years), mean (SD)	66.5 (10.4)	68.9 (10.0)	64.8 (10.4)	< 0.001
BMI, mean (SD)	29.6 (5.9)	29.9 (6.4)	29.4 (5.5)	0.771
Number of risk factors; n (%)				0.522
0	6 (0.9)	2 (0.7)	4 (1.4)	
1–2	158 (24.0)	73 (26.7)	85 (22.0)	
≥ 3	495 (75.1)	198 (72.5)	297 (76.9)	
Cardiovascular risk factors; n (%)				
Diabetes mellitus	218 (33.1)	79 (28.9)	139 (36.0)	0.164
Smoking^b^	344 (52.2)	104 (38.1)	240 (62.2)	< 0.001
Overweight	519 (78.8)	212 (77.7)	307 (79.5)	0.712
Family history	210 (31.9)	98 (35.9)	112 (29.0)	0.164
Hypertension	549 (83.3)	236 (86.5)	313 (81.1)	0.164
Hypercholesterolaemia	366 (55.5)	150 (55.0)	216 (56.0)	0.840
Comorbidities; n (%)				
CAD history	335 (50.8)	114 (41.8)	221 (57.3)	< 0.001
Chronic renal failure	47 (7.1)	16 (5.9)	31 (8.0)	0.494
Respiratory disease	109 (16.5)	47 (17.2)	62 (16.1)	0.777
Peripheral artery disease	61 (9.3)	20 (7.3)	41 (10.6)	0.285
Depression	30 (4.6)	15 (5.5)	15 (3.9)	0.521
Type of anginal symptoms; n (%)				0.930
Typical angina	224 (34.0)	95 (34.8)	129 (33.4)	
Atypical angina	270 (41.0)	110 (40.3)	160 (41.5)	
Non-anginal thoracic constraints	165 (25.0)	68 (24.9)	97 (25.1)	
Symptom severity; n (%)				0.196
CCS-Grade 0	52 (7.9)	17 (6.2)	35 (9.1)	
CCS-Grade 1	110 (16.7)	36 (13.2)	74 (19.2)	
CCS-Grade 2	205 (31.1)	94 (34.4)	111 (28.8)	
CCS-Grade 3	230 (32.9)	99 (36.3)	131 (33.9)	
CCS-Grade 4^c^	62 (9.4)	27 (9.9)	35 (9.1)	
Non-invasive testing; n (%)	237 (36.0)	86 (31.5)	151 (39.1)	0.164
Non-invasive test result^d^; n (%)				0.669
Positive	130 (19.7)	43 (15.8)	87 (22.5)	
Negative	37 (5.6)	15 (5.5)	22 (5.7)	
Unclear	69 (10.5)	28 (10.3)	41 (10.6)	
Pre-test probability; n (%)				< 0.001
Low (< 15%)	14 (2.1)	14 (5.1)	0 (0.0)	
Intermediate (15–85%)	600 (91.0)	259 (94.9)	341 (88.3)	
High (≥ 85%)	45 (6.8)	0 (0.0)	45 (11.7)	
Coronary angiography				
Guideline-adherent	169 (25.6)	53 (19.4)	116 (30.1)	0.002
CAD	424 (64.3)	138 (50.5)	286 (74.1)	< 0.001

*BMI* body mass index; *CAD* coronary artery disease; *CCS* Canadian Cardiovascular Society; *SD* standard deviation

^a^All *p* values of baseline characteristics were adjusted using Benjamini–Hochberg procedure

^b^Ever (current or in the past)

^c^Patients reporting symptoms at rest but without acute coronary syndrome

^d^Result of one man missing

### Sex difference in guideline adherence and association between patient sex and guideline adherence (research question i))

Guideline adherence for CA was lower in women than in men (19.4% vs. 30.1%, difference [95% CI] 0.106 [0.04;0.17], *p* = 0.002, see Table 3).

Regression models identified patient sex as a predictor of guideline adherence. According to Model 1, after adjusting for age, typical angina, diabetes mellitus, non-invasive testing, negative non-invasive test result and positive non-invasive test result, CAs were less likely to be guideline-adherent for women than for men (OR [95% CI] 0.40 [0.23;0.69], *p* = 0.002). Model performance was high (Nagelkerke R^2^ = 0.628).

After excluding non-invasive testing and its results (Model 2), the significant sex difference in guideline adherence remained. When adjusting for age, typical angina, hypertension, CAD history and CCS-Grade 3, CAs were less likely to be guideline-adherent for women than for men (OR [95% CI] 0.44 [0.30;0.66], *p* < 0.001). Compared to Model 1, performance was lower in Model 2 (Nagelkerke R^2^ = 0.114). Table 4 displays the results of the two regression models (see Online Resource, Tables S2: results before Benjamini–Hochberg adjustment and Tables S3: results of the first step of regression).

**Table 4:** Multiple binary logistic regression models for guideline adherence

Independent variable^c,d^	Model 1^a^				Model 2^b^			
	OR	95% CI	*p* value^e^	Nagelkerke R^2^	OR	95% CI	*p* value^e^	Nagelkerke R^2^
Sex	0.40^f^	0.23–0.69	0.002	0.628	0.44^f^	0.30–0.66	< 0.001	0.114
Age (in years)	1.09	1.06–1.12	< 0.001		1.04	1.02–1.06	< 0.001	
Diabetes mellitus	1.76	1.00–3.10	0.049					
Smoking								
Overweight								
Family history								
Hypertension					0.46	0.29–0.74	0.002	
Hypercholesterolaemia								
CAD history					0.58	0.39–0.85	0.008	
Chronic renal failure								
Respiratory disease								
Peripheral artery disease								
Depression								
Typical angina	4.72	2.63–8.47	< 0.001		1.96	1.35–2.86	< 0.001	
Atypical angina								
CCS-Grade 0								
CCS-Grade 1								
CCS-Grade 3					0.64	0.43–0.97	0.036	
CCS-Grade 4^g^								
Non-invasive testing	33.06	15.33–71.29	< 0.001					
Non-invasive test result positive	5.72	2.77–11.78	< 0.001					
Non-invasive test result negative	0.13	0.04–0.40	< 0.001					

*CAD* coronary artery disease; *CCS* Canadian Cardiovascular Society; *CI* confidence interval; *OR* odds ratio

^a^Final model: Chi-square 366.980, *p* < 0.001

^b^Final model: Chi-square 53.215, *p* < 0.001

^c^Redundant variables were excluded: non-anginal thoracic constraints, CCS-Grade 2, non-invasive test result unclear

^d^for sex: male sex as reference category; for all other variables: factor/disease not prevalent as reference category

^e^*p* values were adjusted using Benjamini–Hochberg procedure

^f^Univariate OR: 0.71 (95% CI: 0.38–0.81)

^g^Patients reporting symptoms at rest but without acute coronary syndrome

### Sex differences in predictors of guideline adherence (research question ii))

In Model 1-f for women, five predictors of guideline adherence were derived; these explained up to 63% of the variation in guideline adherence (*p* < 0.001, Nagelkerke R^2^ = 0.634). While diabetes mellitus, a respiratory disease, CCS-Grade 4 and a positive non-invasive test result were associated with a higher probability of guideline-adherent CA, presenting with a CAD history was associated with a lower probability of guideline-adherent CA for women. Similarly, for men, six predictors of guideline adherence were detected and up to 61% of the variation in guideline adherence was explained by Model 1-m (*p* < 0.001, Nagelkerke R^2^ = 0.613). Predictors for men differed from those for women. For men, a higher age, a typical angina, prior non-invasive testing and a positive non-invasive test result were associated with a higher probability of guideline-adherent CA, whereas a negative non-invasive test result and being a smoker were associated with a lower probability of guideline-adherent CA.

After excluding non-invasive testing and its results, the sex differences in predictor models of guideline adherence remained (Model 2-f vs. 2-m). For women, only two predictors were identified, and up to 6.6% of the variation in guideline adherence was explained by Model 2-f (*p* = 0.003, Nagelkerke R^2^ = 0.066). A CAD history and overweight were associated with a lower probability of guideline-adherent CA for women. In contrast, for men, six predictors of guideline adherence were detected, and up to 26% of the variation in guideline adherence was explained by Model 2-m (*p* < 0.001, Nagelkerke R^2^ = 0.26). While higher age and a typical angina were associated with a higher probability of guideline-adherent CA, presenting with hypertension, a CAD history or a CCS-Grade 3 was associated with a lower probability of guideline-adherent CA for men, as was being a smoker. The results of the regression models are presented in Table 5 (see Online Resource, Tables S2: results before Benjamini–Hochberg adjustment and Tables S3: results of the first step of regression).

**Table 5:** Multiple binary logistic regression models for guideline adherence, separated for women and men

	Women				Men			
	OR	95% CI	*p* value^a^	Nagelkerke R^2^	OR	95% CI	*p* value^a^	Nagelkerke R^2^
Independent variable^b^	Model 1-f^c^			0.634	Model 1-m^d^			0.613
Age					1.11	1.07–1.15	< 0.001	
Diabetes mellitus	3.68	1.33–10.21	0.017					
Respiratory disease	3.13	1.06–9.23	0.041					
Smoking					0.48	0.25–0.92	0.032	
Typical angina					8.46	4.02–17.79	< 0.001	
Non-invasive testing					14.19	5.49–36.71	< 0.001	
Non-invasive test result positive	173.27	50.02–600.18	< 0.001		7.94	2.97–21.22	< 0.001	
Non-invasive test result negative					0.22	0.05–0.97	0.046	
CCS-Grade 4^e^	4.40	1.23–15.71	0.028					
CAD history	0.23	0.08–0.68	0.013					

	Model 2-f^f^			0.066	Model 2-m^g^			0.260
Overweight	0.42	0.22–0.81	0.015					
Age					1.07	1.04–1.10	< 0.001	
Smoking					0.55	0.34–0.91	0.024	
Typical angina					3.58	2.15–5.97	< 0.001	
Hypertension					0.30	0.17–0.56	< 0.001	
CAD history	0.48	0.25–0.93	0.032		0.52	0.31–0.86	0.016	
CCS-Grade 3					0.51	0.30–0.89	0.023	

*CAD* coronary artery disease; *CCS* Canadian Cardiovascular Society; *CI* confidence interval; *OR* odds ratio

^a^*p* values were adjusted using Benjamini–Hochberg procedure

^b^Redundant variables were excluded: CCS-Grade 2, non-invasive test result unclear, non-anginal thoracic constraints (men)/atypical angina (women)

^c^Final model: Chi-square 138.094, *p* < 0.001

^d^Final model: Chi square 218.580, *p* < 0.001

^e^Patients reporting symptoms at rest but without acute coronary syndrome

^f^Final model: Chi-square 11.467, *p* = 0.003

^g^Final model: Chi-square 78.062, *p* < 0.001

## Discussion

Based on data from the German ENLIGHT-KHK trial, physicians’ adherence to guideline recommendations for CA use was lower for women with suspected CCS compared to men (19% vs. 30%). CAs were significantly less likely to be guideline-adherent for women than for men; this could not be accounted for by other factors such as age, anginal symptoms, risk factors, comorbidities or non-invasive testing alone (Model 1: OR 0.40, *p* = 0.002; Model 2: OR 0.44, *p* < 0.001).

Analyses confirmed that a model including non-invasive testing and its results (Model 1) explained guideline adherence more accurately than a model based on patient characteristics alone (Model 2). Furthermore, the underlying factors for PTP, i.e. age, sex and typical angina, were predictors in both models. However, diabetes mellitus (Model 1), hypertension and CAD history (Model 2) were identified as additional predictors.

Patient characteristics alone were more effective at explaining guideline adherence for men than for women (Model 2-m: Nagelkerke R^2^ 0.260 vs. Model 2-f: Nagelkerke R^2^ 0.066). Furthermore, while non-invasive testing and its result, age and typical angina were all predictors of guideline adherence for men, only a positive non-invasive test result had a strong impact among women. In addition to this, men’s predictors included smoking, hypertension and CCS-Grade 3 (resulting in a lower probability of guideline adherence), whereas women’s predictors included diabetes mellitus, respiratory disease, CCS-Grade 4 (resulting in a higher probability of guideline adherence) and overweight (resulting in a lower probability of guideline adherence). CAD history was associated with a lower probability of guideline adherence for both sexes.

### Comparison with other studies

Our result that CA use is less likely to be guideline-adherent when treating women with suspected CCS than men is in line with the published evidence. Leung et al. investigated adherence to US guidelines in referrals for CA with different indications in an Australian catheterization laboratory and concluded that women were more likely to undergo non-adherent CA than men (OR 2.67, 95% CI 2.24–3.19, *p* < 0.001) [15]. Further studies found that women with suspected CCS are less likely to be referred for CA [12, 22]. This suggests that women with suspected CCS undergo less CA, and when performed, their CAs are less likely to be guideline-adherent compared to men. This indicates an inappropriate selection of women eligible for a CA across different countries and health systems.

In contrast, sex was not identified as a predictor of adherence to the ESC guidelines on the diagnosis and management of the chronic coronary syndrome in the ENLIGHT-KHK population [18]. One reason for this might be that the European recommendations for CA and non-invasive testing differ from the German recommendations. The GNDMG recommends non-invasive testing for patients with a PTP of 15–85% and direct CA for a PTP of > 85% [4]. Because only men can have a PTP of > 85% and only women can have a PTP of < 15%, the GNDMG recommendations depend on patient sex [4]. In contrast, the European guideline is based on downgraded PTP values (compared to previous values used in the GNDMG) [4, 6]. It recommends non-invasive testing as an initial measure for all patients for whom CAD cannot be excluded by clinical assessment, and thus does not depend on patient sex [6].

### Potential reasons for sex differences in guideline adherence for CA

The lower adherence to the GNDMG when treating women compared to men may result from sex differences in the predictors of guideline adherence (indicating sex differences in physicians’ decision-making process for CA use) caused by various potential reasons.

In our analysis, non-invasive testing and its results (both positive and negative) were predictive of guideline adherence for men, but only a positive test result was predictive for women. This might indicate a less frequent use of non-invasive testing when diagnosing women compared to men. One possible reason might be a lower diagnostic accuracy of some non-invasive tests in women. For example, in addition to the lower diagnostic accuracy for anatomic CAD of stress ECG and MPS compared to other non-invasive tests such as CCTA [25], a stress ECG is less sensitive and less specific in women than in men due to a lower ability to perform physical exertion and hormonal factors. Furthermore, MPS results can be false positive in women, due to breast attenuation or smaller sized hearts [23, 26, 27]. This could result in a reduced confidence in non-invasive testing in women among physicians, even though diagnostic accuracy has been shown to be equivalent in women and men for other non-invasive tests (e.g. CCTA, CMR) [27, 28].

In our analysis, the type of anginal symptoms, age and smoking were only predictors for men. This might indicate that physicians are less likely to use these characteristics for decision-making for CA use in women. One possible explanation for this is the challenges physicians’ face when determining the type of anginal symptoms for choosing an appropriate diagnostic option in women with often non-specific clinical symptoms of CAD (e.g. shortness of breath, fatigue) [28–30]. Furthermore, physicians might not take women’s ages into account for decision-making, because women remain at intermediate PTP when aging that indicates non-invasive testing as an initial measure [4]. In contrast, older age can contribute to a high PTP in men, suggesting a direct CA [4]. In addition to this, smoking may be only considered among men due to physicians’ knowledge that this important CAD risk factor is more prevalent in German men than women [31]. Although several studies have shown that smoking has a more detrimental effect for women than for men [29, 32, 33], physicians might assume a particularly high risk of CCS in male smokers and refer them directly for CA (i.e. non-adherent for men with PTP ≤ 85% [4]).

In our analysis, overweight was only a predictor of guideline adherence for women, indicating that physicians may only use this characteristic for decision-making for CA use in women. One possible reason for this is the challenges related to performing some non-invasive tests in overweight patients, which increase the physicians’ limited trust in non-invasive testing in women. In particular, the accuracy of exercise ECG, stress ECHO, MPS and CCTA is often limited in obese patients due to difficulties in exercising, poor signal to noise ratios and attenuation artefacts [34]. Although stress CMR is less affected by obesity [34], it is rarely available in an outpatient setting. This might lead to a referral of overweight women for direct CA (i.e. non-adherent [4]).

While hypertension and CCS-Grade 3 were associated with a lower probability of guideline adherence for men, diabetes mellitus, a respiratory disease and a CCS-Grade 4 were associated with a higher probability for women in our analysis. This might indicate that physicians assume a high likelihood of CCS in men with risk factors and strong symptoms, thus directly referring them for CA (i.e. non-adherent in men with PTP ≤ 85% [4]). Women with such characteristics, on the other hand, may be suspected of having an intermediate likelihood, and receive non-invasive testing first (i.e. adherent [4]). One reason for this might be that physicians assume a lower overall risk of CCS in women due to the lower prevalence of CAD in women compared with men [35]. This was also observed in our analysis (Table 3).

Our solely patient characteristic-based model included more predictors and could explain more precise guideline adherence for men compared to women (Model 2-m vs. 2-f). This might indicate a greater uncertainty of physicians in the diagnostic workup for women with suspected CCS than for men, corresponding to published evidence. For example, many physicians are unsure whether the standard cardiovascular risk prediction methods are equally effective for both sexes [36]. This is supported by our analysis, which observed a correlation between PTP and CAD for men, but not for women. To some extent, this is likely due to guideline recommendations and risk assessment models being predominantly derived from men and not sufficiently considering specific women’s risk factors and clinical presentation [37–39].

However, the lower predictive power of Model 2-f could also indicate that guideline adherence for women depends on factors that are not considered in this analysis. For example, patient preferences [40, 41] could affect physicians’ decisions on CA use.

### Strengths and limitations

The main strength of this analysis is its prospective and multicentre data basis (i.e. the ENLIGHT-KHK trial).

However, some limitations should be considered when interpreting the results of this analysis. Firstly, since data collection was geared towards the aim of the ENLIGHT-KHK trial, not all of the factors that potentially affect guideline adherence for CA could be investigated. For example, patient preferences, organizational factors (e.g. accessibility and reimbursement of diagnostic procedures) and healthcare provider characteristics (e.g. physicians’ attitudes or knowledge) might have altered the model results [40–42].

Secondly, it was not possible to investigate the influence of different non-invasive test types and their results on guideline adherence separately (because of the low number of tests). However, since guidelines recommend different non-invasive image-guided tests (e.g. stress CMR or CCTA) before a CA [4, 6], this should only have had a slight effect on the main results of this analysis.

Thirdly, since guideline adherence may differ depending on the specific healthcare setting and only nine hospitals were included in the analysis, the generalizability of the results for nationwide and international clinical practice may be limited. However, the study population seems comparable to the German national quality assurance cohort in terms of age, sex and body mass index (e.g. 41% vs. 37% women, 73% vs. 76% aged ≥ 60 years, 79% vs. 72% overweight) [43].

Finally, since all the ENLIGHT-KHK trial patients included in this analysis underwent a CA, it was not possible to evaluate patient-related sex differences in a potential underuse of CA (i.e. patients not receiving a recommended CA).

### Implications and future directions

The observed guideline adherence has some consequences. An analysis based on the same population showed marginal negative clinical consequences (more major adverse cardiac events) and a non-negligible additional expenditure for the German Statutory Health Insurance (SHI) for the observed diagnostic workup compared to the complete, guideline-adherent version [20]. Based on the fact that guideline adherence was lower when treating women than men, the consequences of this might have a higher impact for women than for men.

Efforts are needed to reduce the differences between women and men in the evidence-based use of CA for suspected CCS. A number of different approaches are available for this. Firstly, examining and disseminating sex-specific disease characteristics, such as risk factors, predictors and pathophysiological mechanisms, could enable targeted management for women and men. For example, findings could contribute to more sex-specific guideline recommendations for the diagnostic workup of patients with suspected CCS. Secondly, disseminating evidence on the sex-specific diagnostic accuracy of non-invasive testing might improve acceptance and uptake among physicians of appropriate tests for women. Thirdly, enhancing awareness of CCS in women might ensure prompt and adequate diagnosis. Fourthly, further research could evaluate the association between guideline adherence for CA and other factors that were not investigated in our study (e.g. provider characteristics, structural conditions).

After the ENLIGHT-KHK trail, the GNDMG was updated in 2024. This updated version uses modified PTP values and strengthens the use of CCTA [44]. In addition, outpatient CCTA is reimbursed by the SHI from 2025 [45]. Since these changes might impact the clinical situation (e.g. increase the use of non-invasive tests), a reassessment of guideline adherence and potential sex-specific differences is recommended.

## Conclusion

Based on the ENLIGHT-KHK trial population, women with suspected CCS are less likely to undergo a guideline-adherent CA than men, and predictors of guideline adherence differ noticeably between women and men. Our results contribute to the published evidence and may reflect limited awareness and knowledge of CAD in women among healthcare providers, as well as insufficiently sex-specific guideline recommendations.

## Supplementary Information

Below is the link to the electronic supplementary material.

Supplementary file1 (DOCX 60 KB)

## Data Availability

The datasets generated and/or analysed during the current study are not publicly available due to German data protection regulations (as they contain information that could compromise the privacy of research participants). However, aggregated data can be shared upon reasonable request.

## Abbreviations

ACS: Acute coronary syndrome
CA: Coronary angiography
CAD: Coronary artery disease
CCS: Chronic coronary syndrome
CCS-Grade: Canadian Cardiovascular Society angina grade (classifies severity of angina)
CCTA: Coronary computed tomography angiography
CMR: Cardiac magnetic resonance imaging
ECG: Electrocardiogram
ECHO: Echocardiography
ESC: European Society of Cardiology
GNDMG: German national disease management guideline
MPS: Myocardial perfusion scintigraphy
OR: Odds ratio
PTP: Pre-test probability
SD: Standard deviation
SHI: Statutory health insurance
