# Supplementary material for: Sex differences in guideline adherence for coronary angiography in patients with suspected chronic coronary syndrome in Germany: insights from the ENLIGHT-KHK trial
Source: Clin Res Cardiol. 2025 May 6;114(12):1718–29. doi: 10.1007/s00392-025-02655-y (PMC12708741; doi:10.1007/s00392-025-02655-y)
Supplement: Supplementary file 1 — Supplementary file1 (DOCX 60 KB) [file 392_2025_2655_MOESM1_ESM.docx]

**Sex differences in guideline adherence for coronary angiography in patients with suspected chronic coronary artery disease in Germany: insights from the ENLIGHT-KHK trial - Supplementary Material**

*Clinical Research in Cardiology*

Hannah Kentenich^1*^, Arim Shukri^1^, Dirk Müller^1^, Bastian Wein^2,3^, Oliver Bruder^2,4^, Stephanie Stock^1^, Yana Kampfer^1^

^1^ University of Cologne, Faculty of Medicine and University Hospital Cologne, Institute for Health Economics and Clinical Epidemiology, Gleueler Straße 176-178, 50935 Cologne, Germany

^2^ Elisabeth-Hospital, Contilia Heart and Vascular Centre, Klara-Kopp-Weg 1, 45138 Essen, Germany

^3^ University of Augsburg, Faculty of Medicine, Cardiology, Stenglinstrasse 2, 86156 Augsburg, Germany

^4^ Ruhr University Bochum, Faculty of Medicine, 44801 Bochum, Germany

^*^ hannah.kentenich@uk-koeln.de

Supplementary Text S1: Details on regression analysis

For all regression models, covariates with > 2 categories were transformed into dummy variables. In addition, it was checked for the basic assumptions for conducting logistic regression (i.e., independence of errors, linearity in the logit for any continuous independent variable, lack of strongly influential outliers and absence of multicollinearity or redundancy[1]). All basic assumptions for logistic regression were fulfilled. Stepwise backward regression was conducted by starting with a full model that included all variables and gradually eliminating the least promising ones using the prespecified significance level[1] 0.05.

Supplementary Table S1: Baseline characteristics and coronary angiography results before Benjamini-Hochberg adjustment

| Baseline characteristics of participants | Total  (n=659) | Women  (n=273) | Men  (n=386) | P-value |
| --- | --- | --- | --- | --- |
| Age (years), mean (SD) | 66.5 (10.4) | 68.9 (10.0) | 64.8 (10.4) | <0.001* |
| BMI, mean (SD) | 29.6 (5.9) | 29.9 (6.4) | 29.4 (5.5) | 0.649 |
| Number of risk factors; n (%)  0  1-2  ≥3 | 6 (0.9)  158 (24.0)  495 (75.1) | 2 (0.7)  73 (26.7)  198 (72.5) | 4 (1.4)  85 (22.0)  297 (76.9) | 0.357 |
| Cardiovascular risk factors; n (%)  Diabetes mellitus  Smoking^a^  Overweight  Family history  Hypertension  Hypercholsterolaemia | 218 (33.1)  344 (52.2)  519 (78.8)  210 (31.9)  549 (83.3)  366 (55.5) | 79 (28.9)  104 (38.1)  212 (77.7)  98 (35.9)  236 (86.5)  150 (55.0) | 139 (36.0)  240 (62.2)  307 (79.5)  112 (29.0)  313 (81.1)  216 (56.0) | 0.057  <0.001*  0.562  0.062  0.069  0.796 |
| Comorbidities; n (%)  CAD history  Chronic renal failure  Respiratory disease  Peripheral artery disease  Depression | 335 (50.8)  47 (7.1)  109 (16.5)  61 (9.3)  30 (4.6) | 114 (41.8)  16 (5.9)  47 (17.2)  20 (7.3)  15 (5.5) | 221 (57.3)  31 (8.0)  62 (16.1)  41 (10.6)  15 (3.9) | <0.001*  0.286  0.695  0.150  0.329 |
| Complaints type; n (%)  Typical angina  Atypical angina  Non-anginal thoracic constraints | 224 (34.0)  270 (41.0)  165 (25.0) | 95 (34.8)  110 (40.3)  68 (24.9) | 129 (33.4)  160 (41.5)  97 (25.1) | 0.930 |
| Symptom severity; n (%)  CCS-Grade 0  CCS-Grade 1  CCS-Grade 2  CCS-Grade 3  CCS-Grade 4^b^ | 52 (7.9)  110 (16.7)  205 (31.1)  230 (32.9)  62 (9.4) | 17 (6.2)  36 (13.2)  94 (34.4)  99 (36.3)  27 (9.9) | 35 (9.1)  74 (19.2)  111 (28.8)  131 (33.9)  35 (9.1) | 0.093 |
| Non-invasive test; n (%) | 237 (36.0) | 86 (31.5) | 151 (39.1) | 0.045* |
| Non-invasive test result^c^; n (%)  Positive  Negative  unclear | 130 (19.7)  37 (5.6)  69 (10.5) | 43 (15.8)  15 (5.5)  28 (10.3) | 87 (22.5)  22 (5.7)  41 (10.6) | 0.493 |
| Pre-test probability; n (%)  Low (<15%)  Intermediate (15-85%)  High (≥85%) | 14 (2.1)  600 (91.0)  45 (6.8) | 14 (5.1)  259 (94.9)  0 (0.0) | 0 (0.0)  341 (88.3)  45 (11.7) | <0.001* |
| Coronary angiography | | | | |
| Guideline adherent | 169 (25.6) | 53 (19.4) | 116 (30.1) | 0.002 |
| CAD | 424 (64.3) | 138 (50.5) | 286 (74.1) | <0.001 |

^a^ ever (current or in the past), ^b^ Patients reporting symptoms at rest but without acute coronary syndrome, ^c^ result of one man missing

BMI = Body Mass Index, CAD = Coronary Artery Disease, CCS = Canadian Cardiovascular Society, SD = Standard Deviation

Supplementary Tables S2: Regression results before Benjamini-Hochberg adjustment

S2.1. Multiple binary logistic regression analysis for guideline adherence

|  | Model 1^a^ | | |  |  | Model 2^b^ | | |  |
| --- | --- | --- | --- | --- | --- | --- | --- | --- | --- |
| Variable^c,d^ | OR | 95% CI | P-value | Nagelkerke R^2^ |  | OR | 95% CI | P-value | Nagelkerke R^2^ |
| Sex | 0.40 | 0.23 -0.69 | 0.001 | 0.628 |  | 0.44 | 0.30-0.66 | <0.001 | 0.114 |
| Age (in years) | 1.09 | 1.06-1.12 | <0.001 |  |  | 1.04 | 1.02-1.06 | <0.001 |  |
| Diabetes mellitus | 1.76 | 1.00-3.10 | 0.049 |  |  |  |  |  |  |
| Smoking |  |  |  |  |  |  |  |  |  |
| Overweight |  |  |  |  |  |  |  |  |  |
| Family history |  |  |  |  |  |  |  |  |  |
| Hypertension |  |  |  |  |  | 0.46 | 0.29-0.74 | 0.001 |  |
| Hypercholesterolaemia |  |  |  |  |  |  |  |  |  |
| CAD history |  |  |  |  |  | 0.58 | 0.39-0.85 | 0.005 |  |
| Chronic renal failure |  |  |  |  |  |  |  |  |  |
| Respiratory disease |  |  |  |  |  |  |  |  |  |
| Peripheral artery disease |  |  |  |  |  |  |  |  |  |
| Depression |  |  |  |  |  |  |  |  |  |
| Typical angina | 4.72 | 2.63-8.47 | <0.001 |  |  | 1.96 | 1.35-2.86 | <0.001 |  |
| Atypical angina |  |  |  |  |  |  |  |  |  |
| CCS-Grade 0 |  |  |  |  |  |  |  |  |  |
| CCS-Grade 1 |  |  |  |  |  |  |  |  |  |
| CCS-Grade 3 |  |  |  |  |  | 0.64 | 0.43-0.97 | 0.033 |  |
| CCS-Grade 4^e^ |  |  |  |  |  |  |  |  |  |
| Non-invasive testing | 33.06 | 15.33-71.29 | <0.001 |  |  |  |  |  |  |
| Non-invasive test result positive | 5.72 | 2.77-11.78 | <0.001 |  |  |  |  |  |  |
| Non-invasive test result negative | 0.13 | 0.04-0.40 | <0.001 |  |  |  |  |  |  |

^a^ Final model: chi-square 366.980, p<0.001

^b^ Final model: chi-square 53.215, p<0.001

^c^ Redundant variables were excluded: non-anginal thoracic constraints, CCS-Grade 2, non-invasive test result unclear

^d^ for sex: male sex as reference category; for all other variables: factor/disease not prevalent as reference category

^e^ Patients reporting symptoms at rest but without acute coronary syndrome

CAD = Coronary Artery Disease, CCS = Canadian Cardiovascular Society, CI = Confidence Interval, OR = Odds Ratio

S2.2. Multiple binary logistic regression analysis for guideline adherence, separated for women and men

|  | Women | | |  |  | Men | | |  |
| --- | --- | --- | --- | --- | --- | --- | --- | --- | --- |
|  | OR | 95% CI | P-value | Nagelkerke R^2^ |  | OR | 95% CI | P-value | Nagelkerke R^2^ |
| Variable^a,b^ | **Model 1-f^c^** | | | 0.634 |  | **Model 1-m^d^** | | | 0.613 |
| Age (in years) |  |  |  |  |  | 1.11 | 1.07-1.15 | <0.001 |  |
| Diabetes mellitus | 3.68 | 1.33-10.21 | 0.012 |  |  |  |  |  |  |
| Respiratory disease | 3.13 | 1.06-9.23 | 0.038 |  |  |  |  |  |  |
| Smoking |  |  |  |  |  | 0.48 | 0.25-0.92 | 0.027 |  |
| Typical angina |  |  |  |  |  | 8.46 | 4.02-17.79 | <0.001 |  |
| Non-invasive testing |  |  |  |  |  | 14.19 | 5.49-36.71 | <0.001 |  |
| Non-invasive test result positive | 173.27 | 50.02-600.18 | <0.001 |  |  | 7.94 | 2.97-21.22 | <0.001 |  |
| Non-invasive test result negative |  |  |  |  |  | 0.22 | 0.05-0.97 | 0.045 |  |
| CCS-Grade 4^e^ | 4.40 | 1.23-15.71 | 0.023 |  |  |  |  |  |  |
| CAD history | 0.23 | 0.08-0.68 | 0.008 |  |  |  |  |  |  |
| Variable^a,b^ | **Model 2-f^e^** | | | 0.066 |  | **Model 2-m^g^** | | | 0.260 |
| Overweight | 0.42 | 0.22-0.81 | 0.010 |  |  |  |  |  |  |
| Age (in years) |  |  |  |  |  | 1.07 | 1.04-1.10 | <0.001 |  |
| Smoking |  |  |  |  |  | 0.55 | 0.34-0.91 | 0.019 |  |
| Typical Angina |  |  |  |  |  | 3.58 | 2.15-5.97 | <0.001 |  |
| Hypertension |  |  |  |  |  | 0.30 | 0.17-0.56 | <0.001 |  |
| CAD history | 0.48 | 0.25-0.93 | 0.028 |  |  | 0.52 | 0.31-0.86 | 0.011 |  |
| CCS-Grade 3 |  |  |  |  |  | 0.51 | 0.30-0.89 | 0.017 |  |

^a^ Redundant variables were excluded: CCS-Grade 2, non-invasive test result unclear, non-anginal thoracic constraints (men)/atypical angina (women)

^b^ for all variables: factor/disease not prevalent as reference category

^c^ Final model: chi-square 138.094, p<0.001

^d^ Final model: chi-square 218.580, p<0.001

^e^ Patients reporting symptoms at rest but without acute coronary syndrome

^f^ Final model: chi-square 11.467, p=0.003

^g^ Final model: chi-square 78.062, p<0.001

CAD = Coronary Artery Disease, CCS = Canadian Cardiovascular Society, CI = Confidence Interval, OR = Odds Ratio

Supplementary Tables S3: Results of the first step of regression

S3.1. Multiple binary logistic backwards regression analysis for guideline adherence

|  | Model 1 | | |  |  | Model 2 | | |  |
| --- | --- | --- | --- | --- | --- | --- | --- | --- | --- |
| Variable^a,b^ | OR | 95% CI | P-value | Nagelkerke R^2^ |  | OR | 95% CI | P-value | Nagelkerke R^2^ |
| Sex | 0.35 | 0.19-0.64 | <0.001 | 0.651 |  | 0.40 | 0.26-0.61 | <0.001 | 0.136 |
| Age (in years) | 1.08 | 1.05-1.12 | <0.001 |  |  | 1.03 | 1.01-1.05 | 0.008 |  |
| Diabetes mellitus | 1.85 | 1.01-3.38 | 0.046 |  |  | 1.24 | 0.83-1.87 | 0.297 |  |
| Smoking | 0.59 | 0.33-1.08 | 0.085 |  |  | 0.65 | 0.43-0.99 | 0.043 |  |
| Overweight | 0.81 | 0.43-1.54 | 0.515 |  |  | 0.78 | 0.50-1.23 | 0.291 |  |
| Family history of CAD | 0.88 | 0.48-1.61 | 0.679 |  |  | 1.14 | 0.75-1.74 | 0.537 |  |
| Hypertension | 0.83 | 0.40-1.73 | 0.620 |  |  | 0.48 | 0.29-0.78 | 0.003 |  |
| Hypercholesterolaemia | 0.79 | 0.45-1.39 | 0.414 |  |  | 1.15 | 0.78-1.70 | 0.480 |  |
| CAD history | 0.99 | 0.55-1.79 | 0.963 |  |  | 0.57 | 0.38-0.85 | 0.006 |  |
| Chronic renal failure | 0.79 | 0.26-2.45 | 0.686 |  |  | 1.09 | 0.53-2.27 | 0.814 |  |
| Respiratory disease | 1.26 | 0.59-2.69 | 0.547 |  |  | 1.39 | 0.83-2.31 | 0.207 |  |
| Peripheral artery disease | 2.08 | 0.84-5.15 | 0.114 |  |  | 1.05 | 0.55-2.02 | 0.883 |  |
| Depression | 2.84 | 0.74-10.85 | 0.128 |  |  | 1.17 | 0.47-2.95 | 0.733 |  |
| Typical angina | 3.33 | 1.53-7.25 | 0.002 |  |  | 1.80 | 1.08-2.99 | 0.024 |  |
| Atypical angina | 0.51 | 0.24-1.08 | 0.079 |  |  | 0.83 | 0.50-1.37 | 0.468 |  |
| CCS-Grade 0 | 1.58 | 0.53-4.73 | 0.417 |  |  | 1.34 | 0.65-2.76 | 0.435 |  |
| CCS-Grade 1 | 0.56 | 0.23-1.36 | 0.199 |  |  | 0.73 | 0.40-1.32 | 0.293 |  |
| CCS-Grade 3 | 0.92 | 0.47-1.81 | 0.803 |  |  | 0.63 | 0.39-1.00 | 0.052 |  |
| CCS-Grade 4^c^ | 1.93 | 0.78-4.82 | 0.157 |  |  | 1.17 | 0.62-2.24 | 0.628 |  |
| Non-invasive testing | 37.61 | 16.53-85.55 | <0.001 |  |  | - | - | - |  |
| Non-invasive test result positive | 6.43 | 2.97-13.92 | <0.001 |  |  | - | - | - |  |
| Non-invasive test result negative | 0.15 | 0.05-0.46 | 0.001 |  |  | - | - | - |  |

^a^ Redundant variables were excluded: non-anginal thoracic constraints, CCS-Grade 2, non-invasive test result unclear

^b^ for sex: male sex as reference category; for all other variables: factor/disease not prevalent as reference category

^c^ Patients reporting symptoms at rest but without acute coronary syndrome

CAD = Coronary Artery Disease, CCS = Canadian Cardiovascular Society, CI = Confidence Interval, OR = Odds Ratio

S3.2. Multiple binary logistic backwards regression analysis for guideline adherence in women

|  | Model 1-f | | | |  | |  | | Model 2-f | | | |  | |
| --- | --- | --- | --- | --- | --- | --- | --- | --- | --- | --- | --- | --- | --- | --- |
| Variable^a,b^ | OR | 95% CI | P-value | Nagelkerke R^2^ | |  | | OR | | 95% CI | P-value | Nagelkerke R^2^ | |  |
| Age (in years) | 1.09 | 0.95-1.26 | 0.226 | 0.890 | |  | | 0.96 | | 0.93-1.00 | 0.049 | 0.182 | |  |
| Diabetes mellitus | 51.15 | 2.41-1087.54 | 0.012 |  | |  | | 2.32 | | 1.11-4.84 | 0.026 |  | |  |
| Smoking | 1.31 | 0.2-8.76 | 0.781 |  | |  | | 0.92 | | 0.43-1.98 | 0.826 |  | |  |
| Overweight | 0.16 | 0.02-1.27 | 0.082 |  | |  | | 0.29 | | 0.14-0.62 | 0.001 |  | |  |
| Family history of CAD | 0.72 | 0.10-5.09 | 0.743 |  | |  | | 0.83 | | 0.40-1.72 | 0.618 |  | |  |
| Hypertension | 0.74 | 0.09-6.32 | 0.785 |  | |  | | 0.83 | | 0.33-2.08 | 0.689 |  | |  |
| Hypercholesterolaemia | 0.71 | 0.13-3.83 | 0.693 |  | |  | | 0.98 | | 0.50-1.94 | 0.959 |  | |  |
| CAD history | 0.52 | 0.08-3.55 | 0.501 |  | |  | | 0.49 | | 0.23-1.04 | 0.064 |  | |  |
| Chronic renal failure | 2.69 | 0.00-6.03E+40 | 0.983 |  | |  | | 0.47 | | 0.09-2.52 | 0.379 |  | |  |
| Respiratory disease | 1.18 | 0.13-10.74 | 0.884 |  | |  | | 1.90 | | 0.79-4.58 | 0.155 |  | |  |
| Peripheral artery disease | 2.32 | 0.07-75.95 | 0.637 |  | |  | | 0.26 | | 0.05-1.36 | 0.111 |  | |  |
| Depression | 23882145.48 | - | 0.993 |  | |  | | 1.78 | | 0.48-6.56 | 0.385 |  | |  |
| Typical angina | 0.89 | 0.09-8.77 | 0.920 |  | |  | | 0.62 | | 0.28-1.39 | 0.245 |  | |  |
| Non-anginal thoracic constraints | 20.54 | 0.66-642.83 | 0.085 |  | |  | | 1.01 | | 0.45-2.28 | 0.984 |  | |  |
| CCS-Grade 0 | 100.06 | 0.20-51085.52 | 0.148 |  | |  | | 3.07 | | 0.85-11.05 | 0.086 |  | |  |
| CCS-Grade 1 | 0.62 | 0.05-8.08 | 0.715 |  | |  | | 0.70 | | 0.22-2.26 | 0.555 |  | |  |
| CCS-Grade 3 | 3.55 | 0.38-33.13 | 0.266 |  | |  | | 1.13 | | 0.50-2.58 | 0.769 |  | |  |
| CCS-Grade 4^c^ | 87.34 | 1.46-5234.99 | 0.032 |  | |  | | 2.63 | | 0.89-7.80 | 0.081 |  | |  |
| Non-invasive testing | 2.67E+16 | - | 0.989 |  | |  | | - | | - | - |  | |  |
| Non-invasive test result positive | 17.58 | 2.38-129.88 | 0.005 |  | |  | | - | | - | - |  | |  |
| Non-invasive test result negative | 0.00 | 0.00-0.18 | 0.007 |  | |  | | - | | - | - |  | |  |

^a^ Redundant variables were excluded: CCS-Grade 2, non-invasive test result unclear, non-anginal thoracic constraints (men)/atypical angina (women)

^b^ for all variables: factor/disease not prevalent as reference category

^c^ Patients reporting symptoms at rest but without acute coronary syndrome

CAD = Coronary Artery Disease, CCS = Canadian Cardiovascular Society, CI = Confidence Interval, OR = Odds Ratio

S3.3. Multiple binary logistic backwards regression analysis for guideline adherence in men

|  | Model 1-m | | |  |  | Model 2-m | | |  |
| --- | --- | --- | --- | --- | --- | --- | --- | --- | --- |
| Variable^a,b^ | OR | 95% CI | P-value | Nagelkerke R^2^ |  | OR | 95% CI | P-value | Nagelkerke R^2^ |
| Age (in years) | 1.11 | 1.07-1.16 | <0.001 | 0.639 |  | 1.08 | 1.05-1.11 | <0.001 | 0.284 |
| Diabetes mellitus | 1.50 | 0.71-3.14 | 0.288 |  |  | 1.09 | 0.63-1.89 | 0.770 |  |
| Smoking | 0.40 | 0.20-0.81 | 0.011 |  |  | 0.49 | 0.29-0.82 | 0.007 |  |
| Overweight | 1.10 | 0.47-2.58 | 0.823 |  |  | 1.41 | 0.74-2.68 | 0.295 |  |
| Family history of CAD | 1.58 | 0.73-3.45 | 0.247 |  |  | 1.70 | 0.94-3.05 | 0.077 |  |
| Hypertension | 0.53 | 0.22-1.29 | 0.159 |  |  | 0.27 | 0.14-0.52 | <0.001 |  |
| Hypercholesterolaemia | 1.08 | 0.53-2.20 | 0.835 |  |  | 1.34 | 0.78-2.28 | 0.287 |  |
| CAD history | 0.88 | 0.41-1.87 | 0.734 |  |  | 0.46 | 0.27-0.80 | 0.006 |  |
| Chronic renal failure | 1.23 | 0.35-4.26 | 0.746 |  |  | 1.45 | 0.57-3.68 | 0.440 |  |
| Respiratory disease | 0.97 | 0.38-2.46 | 0.946 |  |  | 1.26 | 0.63-2.49 | 0.515 |  |
| Peripheral artery disease | 2.11 | 0.75-5.89 | 0.156 |  |  | 1.21 | 0.52-2.83 | 0.655 |  |
| Depression | 1.54 | 0.19-12.77 | 0.691 |  |  | 1.15 | 0.26-5.08 | 0.858 |  |
| Typical angina | 6.69 | 2.46-18.22 | <0.001 |  |  | 3.26 | 1.58-6.74 | 0.001 |  |
| Atypical angina | 0.55 | 0.22-1.41 | 0.215 |  |  | 0.77 | 0.38-1.54 | 0.458 |  |
| CCS-Grade 0 | 1.07 | 0.27-4.31 | 0.926 |  |  | 1.02 | 0.38-2.75 | 0.976 |  |
| CCS-Grade 1 | 0.48 | 0.16-1.43 | 0.188 |  |  | 0.81 | 0.37-1.76 | 0.590 |  |
| CCS-Grade 3 | 0.55 | 0.23-1.27 | 0.161 |  |  | 0.46 | 0.24-0.88 | 0.019 |  |
| CCS-Grade 4^c^ | 1.33 | 0.41-4.26 | 0.635 |  |  | 0.90 | 0.36-2.24 | 0.826 |  |
| Non-invasive testing | 16.60 | 5.89-46.78 | <0.001 |  |  | - | - | - |  |
| Non-invasive test result positive | 6.65 | 2.32-19.04 | <0.001 |  |  | - | - | - |  |
| Non-invasive test result negative | 0.29 | 0.06-1.36 | 0.117 |  |  | - | - | - |  |

^a^ Redundant variables were excluded: CCS-Grade 2, non-invasive test result unclear, non-anginal thoracic constraints (men)/atypical angina (women)

^b^ for all variables: factor/disease not prevalent as reference category

^c^ Patients reporting symptoms at rest but without acute coronary syndrome

CAD = Coronary Artery Disease, CCS = Canadian Cardiovascular Society, CI = Confidence Interval, OR = Odds Ratio

**References**

1. Stoltzfus JC. Logistic Regression: A Brief Primer. *Acad Emerg Med*. 2011;18(10):1099-1104. doi:10.1111/j.1553-2712.2011.01185.x
